# Comparative analysis of late gadolinium enhancement assessment techniques for monitoring fibrotic changes in myocarditis follow-up

**DOI:** 10.1007/s00330-024-10756-x

**Published:** 2024-05-04

**Authors:** Mihály Károlyi, Malgorzata Polacin, Márton Kolossváry, Justyna M. Sokolska, Ioannis Matziris, Lucas Weber, Hatem Alkadhi, Robert Manka

**Affiliations:** 1 Diagnostic and Interventional Radiology, University Hospital Zurich, University of Zurich, Zurich, Switzerland; 2 Institute for Biomedical Engineering, University and ETH Zurich, Zurich, Switzerland; 3 Gottsegen National Cardiovascular Center, Budapest, Hungary; 4 Physiological Controls Research Center, University Research and Innovation Center, Óbuda University, Budapest, Hungary; 5 Department of Cardiology, University Heart Center, University Hospital Zurich, University of Zurich, Zurich, Switzerland; 6 Department of Heart Diseases, Wroclaw Medical University, Wroclaw, Poland; 7 Department of Radiology, Cantonal Hospital Winterthur, Winterthur, Switzerland

**Keywords:** Follow-up studies, Magnetic resonance imaging, Myocarditis

## Abstract

**Objectives:**

To compare the repeatability and interrelation of various late gadolinium enhancement (LGE) assessment techniques for monitoring fibrotic changes in myocarditis follow-up.

**Materials and methods:**

LGE extent change between baseline and 3-month cardiovascular magnetic resonance (CMR) was compared in patients with acute myocarditis using the full width at half maximum (FWHM), gray-scale thresholds at 5 and 6 standard deviations (SD5 and SD6), visual assessment with threshold (VAT) and full manual (FM) techniques. In addition, visual presence score (VPS), visual transmurality score (VTS), and a simplified visual change score (VCS) were assessed. Intraclass-correlation (ICC) was used to evaluate repeatability, and methods were compared using Spearman’s correlation.

**Results:**

Forty-seven patients (38 male, median age: 27 [IQR: 21; 38] years) were included. LGE extent change differed among quantitative techniques (*p* < 0.01), with variability in the proportion of patients showing LGE change during follow-up (FWHM: 62%, SD5: 74%, SD6: 66%, VAT: 43%, FM: 60%, VPS: 53%, VTS: 77%, VCS: 89%). Repeatability was highest with FWHM (ICC: 0.97) and lowest with SD5 (ICC: 0.89). Semiquantitative scoring had slightly lower values (VPS ICC: 0.81; VTS ICC: 0.71). VCS repeatability was excellent (ICC: 0.93). VPS and VTS correlated with quantitative techniques, while VCS was positively associated with VPS, VTS, VAT, and FM, but not with FWHM, SD5, and SD6.

**Conclusion:**

FWHM offers the least observer-dependent LGE follow-up after myocarditis. VPS, VTS, and VCS are practical alternatives, showing reliable correlations with quantitative methods. Classification of patients exhibiting either stable or changing LGE relies on the assessment technique.

**Clinical relevance statement:**

This study shows that LGE monitoring in myocarditis is technique-dependent; the FWHM method yields the most consistent fibrotic tracking results, with scoring-based techniques as reliable alternatives.

**Key Points:**

- *Recognition of fibrotic changes during myocarditis follow-up is significantly influenced by the choice of the quantification technique employed.*
- *The FWHM technique ensures highly repeatable tracking of myocarditis-related LGE changes.*
- *Segment-based visual scoring and the simplified visual change score offer practical, reproducible alternatives in resource-limited settings.*

## Introduction

Acute myocarditis presents with diverse clinical manifestations. While most patients recover fully, a subset faces severe complications, such as heart failure and cardiovascular death [1]. Identifying individuals at risk of unfavorable outcomes is crucial for tailoring effective therapeutic interventions.

In recent years, diagnostic approaches for myocarditis have evolved significantly. While an endomyocardial biopsy is preferred for cases involving shock or severe arrhythmias, cardiovascular magnetic resonance imaging (CMR) has become the primary diagnostic tool for clinically stable patients with suspected acute myocarditis, recommended as a first-line evaluation for cardiomyopathies (Class I, Level B recommendation) [2]. CMR, especially with late gadolinium enhancement (LGE), offers a robust method to confirm myocyte injury during the acute phase and reliably assess fibrotic changes within affected myocardial regions over the course of inflammation [3, 4]. The persistence of the LGE postacute phase correlates with adverse outcomes, while its absence at follow-up indicates full recovery [5, 6]. Despite the importance of follow-up CMR examinations for disease monitoring and prognostication, current guidelines lack consensus on the optimal LGE assessment technique after the acute phase of myocarditis, representing an active area of investigation [7, 8].

Recently, there has been a growing interest in applying quantitative methods to LGE in myocarditis, akin to their routine use for other cardiomyopathies. These methods often involve gray-scale thresholds combined with semiautomated or fully manual techniques, providing a detailed yet labor-intensive approach to LGE quantification [9, 10]. Simpler, semiquantitative methods, utilizing segment-based scoring systems, remain widely used for LGE assessment [11, 12]. Experienced clinicians may even visually discern significant changes in LGE patterns and extent throughout the entire left ventricular (LV) myocardium, potentially bypassing the need for intricate quantitative analyses. This approach can benefit from clinical intuition and the integration of contextual information into the evaluation process. Limited data exist on directly comparing quantitative and semiquantitative methods for measuring LGE extent in acute myocarditis at a single time point, while their performance in LGE follow-up assessment remains mostly unknown [13].

Thus, our objective was to evaluate the performance and interchangeability of various techniques for assessing LGE changes postacute myocarditis. Our study hypothesized that semiquantitative assessment techniques are as reproducible as quantitative methods in evaluating LGE changes during myocarditis, and they are interchangeable. To address this hypothesis, we introduced a simplified visual assessment method, in addition to segment-based methods, for a direct comparison of LGE changes across the entire LV myocardium, referred to as the ‘visual change score.’

## Materials and methods

### Study population

**Fig. 1:**
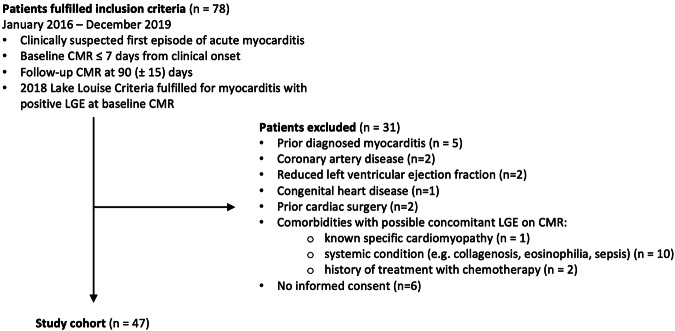
Study flowchart

The study had institutional review board approval, followed the Declaration of Helsinki principles. Patients were retrospectively selected from a myocarditis registry at our institution, with retroactive written informed consent according to institution regulations. Consecutive patients were included, who underwent baseline CMR within 7 days of the initial clinical episode and follow-up CMR at 90 (± 15) days, with a study period between January 2016 and December 2019. All participants were referred to our center with a first episode of suspected acute myocarditis. Patients underwent a diagnostic work-up in line with the latest guidelines of the European Society of Cardiology, including a 12-lead electrocardiogram at presentation, and met the updated Lake Louise Criteria for a CMR-based diagnosis of acute myocarditis [7, 8]. Inclusion criteria required positive LGE on baseline CMR. Coronary artery disease was ruled out in all participants either through invasive coronary angiography, CT coronary angiography, or based on a low pretest probability (< 30 years of age). Medical records were scrutinized for underlying comorbidities that could potentially impact LV myocardium, leading to the exclusion of patients with a history of prior myocarditis, congenital heart disease, cardiac surgery, reduced LV ejection fraction, known cardiomyopathies, and systemic conditions with potential myocardial involvement, such as collagen disorders, eosinophilic disorders, sepsis, tuberculosis, or malignancies undergoing chemotherapy. The study population is outlined in Fig. 1.

### CMR protocol

The studies utilized magnetic resonance scanners, either a 1.5 Tesla (Achieva, Philips Medical Systems) or a 3.0 Tesla (Skyra, Siemens), equipped with dedicated phased array coils for cardiac examinations. All acquisitions were breath-held in end-expiration. Cine-balanced steady-state free precession images assessed cardiac function and geometry in standard orientations. Native T1 and T2 Mapping, along with T2-weighted images of the LV with and without fat suppression, were obtained in short axis. LGE images were acquired 10 minutes postadministration of 0.2 mmoL/kg gadobutrol (Gadovist, Bayer Schering Pharma), following standard orientations. The optimal inversion time for LGE was determined individually using an inversion time scout sequence, ranging between 190 and 270 ms. For 1.5 Tesla CMR studies, a three-dimensional (3D) LGE sequence was employed (field of view 350 × 350 mm^2^, matrix dimensions of 256 × 256, repetition time/echo time of 3.6/1.8 ms, a flip angle of 15°, in-plane resolution of 1.5 × 1.5 mm^2^, and a slice thickness of 8 mm). At 3.0 Tesla, a two-dimensional (2D) Phase-Sensitive Inversion-Recovery sequence was used (field of view 330 × 330 mm^2^, matrix dimensions of 192 × 256, repetition time/echo time of 663/2 ms, a flip angle of 20°, in-plane resolution of 1.3 × 1.3 mm^2^, and a slice thickness of 8 mm). The CMR diagnosis of myocarditis was established based on regional or global myocardial edema, defined by increased native T2-signal or T2-mapping values, along with evidence of myocardial injury indicated by elevated native T1-mapping values, increased extracellular volume, or positive LGE.

### LGE data analysis

LGE involvement in the LV was assessed using commercial postprocessing software (Intellispace Portal, Version 10, Philips Healthcare). The quantitative evaluation involved manually delineating LV endo- and epicardial boundaries on short-axis LGE images. Subsequently, various clinically available LGE quantification techniques were applied. These included semiautomated methods, where regions of interest (ROIs) were initially placed in both non-enhanced areas and the seemingly normal remote myocardium. In the next step, the hyper-enhanced myocardial regions were segmented in several ways: automatically using the full width at half maximum (FWHM) method or relative to gray-scale thresholds set at predefined values, 5 and 6 standard deviations above the mean signal intensity for the remote myocardial tissue (referred to as the 5 SD and 6 SD techniques), or by manually adjusting the threshold by the reader to achieve the best match (referred to as visual assessment with a user-defined threshold, VAT). Lastly, a full manual (FM) quantification method was employed, where the reader manually delineated the extent of LGE on each LV slice. The extent of LGE was calculated as a percentage relative to the entire LV myocardial volume. Change in LGE extent during follow-up was defined as a ≥ 1% difference in LGE volume between baseline and follow-up relative to the entire LV volume. This cut-off was based on the previous prognostic study of Aquaro et al, who used the same threshold, and a significant LGE change from 6.2 [IQR: 2; 10] % to 4.1 [IQR: 2; 8] % was found over 6 months follow-up [6].

For semiquantitative LGE assessment, the 17-segment model of the American Heart Association was applied to the entire LV myocardium [14]. LGE presence and transmurality were coded (0 = no LGE, 1 = < 25%, 2 = 26–50%, 3 = 51–75%, 4 = 76–100%), and visual presence score (VPS) and visual transmurality score (VTS) were calculated, each multiplied by the number of affected myocardial segments, resulting in VPS (0–17) and VTS (0–68). Change in LGE extent was defined as the change of VPS or VTS score during follow-up.

For a simplified assessment of LGE change, baseline and follow-up short-axis LGE images were displayed side by side. Readers estimated the percentage LGE difference of the entire LV myocardium, consolidated into a five-point Likert scale: 0 = no change, 1 = 1–25%, 2 = 26–50%, 3 = 51–75%, and 4 = 76–100%, termed the ‘visual change score’ (VCS). Positive values denote LGE progression, while negative values indicate regression.

Baseline and follow-up CMR studies were independently evaluated by three observers (M.K., M.P., J.M.S.) with over 5 years of CMR experience and Level 3 certification from the European Association of Cardiovascular Imaging or equivalent authority. Consensus readings were used to estimate the correlation between each technique. To test interobserver reliability, one observer (M.K.) repeated measurements with a 6-month interval.

### Statistical analysis

Continuous variables are presented as median and interquartile range (IQR). Categorical variables are expressed as counts and percentages. The Wilcoxon signed-rank test compared patient characteristics between baseline and follow-up for continuous variables, and the McNemar test compared categorical data. Friedman’s test assessed the extent of LGE among different quantitative methods. Spearman’s correlation evaluated the relationship between LGE assessment techniques, with correlation coefficients (ρ) indicating a very strong (≥ 0.70), strong (0.40 to < 0.70), moderate (0.30 to < 0.40), weak (0.20 to < 0.30), and no or negligible (0.01 to < 0.20) relationship [15, 16]. Intra- and interobserver repeatability were assessed using intraclass correlation (ICC), categorized as poor (< 0.5), moderate (0.5 to < 0.75), good (0.75 to < 0.9), or excellent (0.9–1.0) agreement [17]. A 2-sided *p* < 0.05 was considered statistically significant. Statistical analyses were performed using SPSS (version 23).

## Results

### Baseline characteristics

**Fig. 2:**
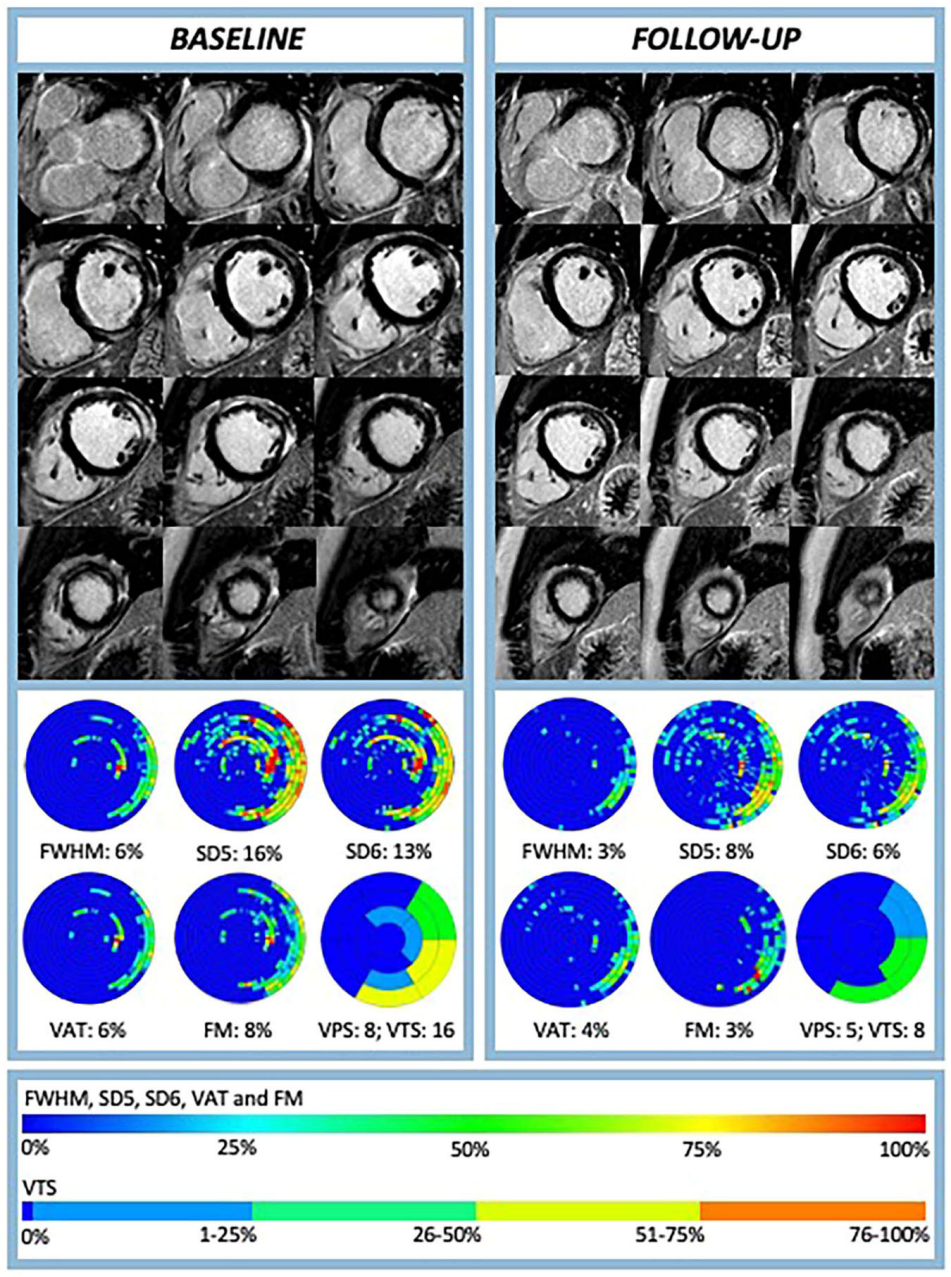
Example of LGE assessment using various quantitative techniques and visual scoring. Cardiovascular magnetic resonance images at baseline and 3-month follow-up of a 35-year-old male with acute myocarditis. Upper panels show LGE images in short-axis orientation, while lower panels present bulls-eye views with quantitative and visual scores. The color scale represents the LGE extent. Changes in LGE extent (percentage, %) from baseline to follow-up: FWHM 6% to 3%, SD5 16% to 8%, SD6 13% to 6%, VAT 6% to 4%, FM 8% to 3%. VPS decreased from 8 to 5, and VTS reduced from 16 to 8, corresponding a VCS of -3, indicating a 51–75% reduction in LGE

The study included 47 patients meeting specific criteria (38 male, median age 27 [IQR: 21; 38] years). Baseline patient characteristics are detailed in Table 1, and diagnostic parameters are summarized in Table 2. Figure 2 illustrates an example of LGE follow-up assessment using various techniques.

**Table 1:** Patient characteristics

	*n* = 47
Baseline characteristics	
Age (years), median [IQR]	27 [21; 38]
Male, *n* (%)	38 (81)
BMI (kg/m^2^), median [IQR]	26 [23; 31]
Cardiovascular risk factors	
Hypertension, *n* (%)	4 (9)
Diabetes, *n* (%)	2 (4)
Hyperlipidemia, *n* (%)	3 (6)
Smoking history, *n* (%)	22 (47)
Active smoker, *n* (%)	17 (36)
Family risk of CAD, *n* (%)	11 (23)
Symptoms at presentation	
Chest pain, *n* (%)	40 (85)
Dyspnea, *n* (%)	8 (17)
Palpitation, *n* (%)	9 (19)
Dizziness or syncope, *n* (%)	5 (11)
Fever, *n* (%)	13 (27)
Recent respiratory infection or flu, *n* (%)	20 (43)
Recent gastrointestinal infection, *n* (%)	12 (25)
Recent other infection, *n* (%)	4 (4)
Timing of examinations	
Admission to baseline CMR (days), median [IQR]	2 (1–3)
Baseline to follow-up CMR (days), median [IQR]	92 [83; 103]

Data as median and interquartile range [IQR] or count and percentage (%), as appropriate

*BMI* body mass index, *CAD* coronary artery disease

**Table 2:** Routine diagnostic parameters at baseline and follow-up

*n* = 47	Baseline	Follow-up		*p* value
Laboratory parameter				
CRP (mg/mL), median [IQR]	23 [6; 60]		1.2 [0.6; 2]	< 0.01
CK (U/L), median [IQR]	219 [98; 426]		114 [88; 158]	< 0.01
Hs-TnT (ng/L), median [IQR]	300 [133; 843]		5 [4; 8]	< 0.01
Myoglobin (µg/L), median [IQR]	35 [23; 75]		30 [24; 39]	0.02
NT-proBNP (ng/L), median [IQR]	176 [71; 635]		25 [13; 40]	< 0.01
ECG parameter				
Nonsinus rhythm, *n* (%)	1 (2)		1 (2)	1.00
Ventricular extrasystole, *n* (%)	3 (6)		2 (4)	1.00
Atrioventricular-block, *n* (%)	1 (2)		1 (2)	1.00
Fascicular-block, *n* (%)	2 (4)		5 (11)	0.08
> 0.5 mm PQ depression, *n* (%)	2 (4)		1 (2)	1.00
> 1 mm ST-segment elevation, *n* (%)	14 (30)		2 (4)	< 0.01
> 1 mm ST-segment depression, *n* (%)	3 (6)		1 (2)	0.50
T-inversion, *n* (%)	17 (36)		13 (28)	0.45
Pathological QRS-T angle (> 100^o^), *n* (%)	4 (9)		1 (2)	0.50
Wide QRS complex (>120 ms), *n* (%)	2 (4)		0 (0)	0.50
Prolonged QTc interval (> 420 ms), *n* (%)	18 (38)		8 (17)	0.01
CMR parameter				
LVEDDi (mm/m^2^), median [IQR]	27 [25; 29]		27 [25; 28]	0.26
Septum thickness (mm), median [IQR]	8 [8; 9]		8 [8; 9]	0.91
Lateral wall thickness (mm), median [IQR]	8 [7; 9]		8 [7; 8]	0.08
LVEF (%), median [IQR]	56 [54; 61]		56 [55; 59]	0.92
LVEDVi (mL/m^2^), median [IQR]	84 [73; 96]		85 [73; 93]	0.18
LVESVi (mL/m^2^), median [IQR]	36 [30; 43]		37 [39; 40]	0.58
LV mass index (g/m^2^), median [IQR]	57 [49; 69]		50 [44; 58]	< 0.01
RVEF (%), median [IQR]	59 [55; 63]		59 [57; 63]	0.99
RVEDVi (mL/m^2^), median [IQR]	81 [70; 91]		80 [66; 87]	0.56
RVESVi (mL/m^2^), median [IQR]	33 [28; 39]		32 [24; 39]	0.58

Data as median and interquartile range [IQR] or count and percentage (%), as appropriate

*CRP* C-reactive protein, *CK* creatine kinase, *Hs-TnT* high sensitive troponine, *NT-proBNP* N-terminal pro B-type natriuretic peptide, *LVEDDi* left ventricular end-diastolic diameter index, *LVEF* left ventricular ejection fraction, *LVEDVi* left ventricular end-diastolic volume index, *LVESVi* left ventricular end-systolic volume index, *RVEF* right ventricular ejection fraction, *RVEDVi* right ventricular end-diastolic volume index, *RVESVi* right ventricular end-systolic volume index

### LGE findings between baseline and at follow-up

**Fig. 3:**
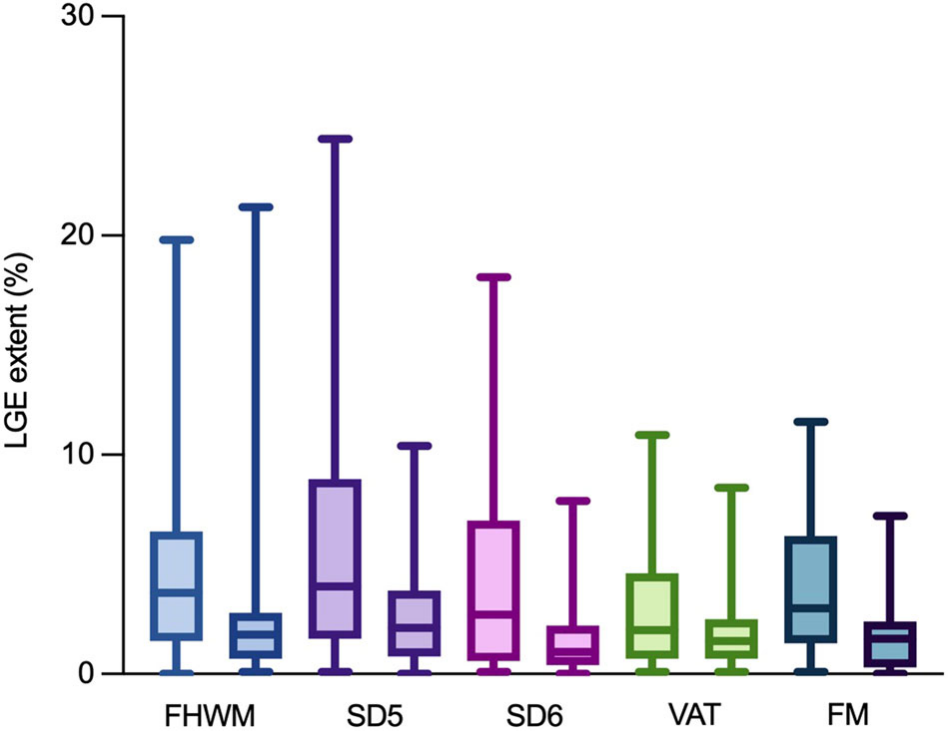
LGE extent at baseline and follow-up CMR with various quantitative techniques. The chart shows LGE extent with various quantitative methods. Columns depict baseline and follow-up LGE percentages. Horizontal lines denote max and min values. Colored boxes highlight data between the first and third quartiles, with lines indicating medians

**Fig. 4:**
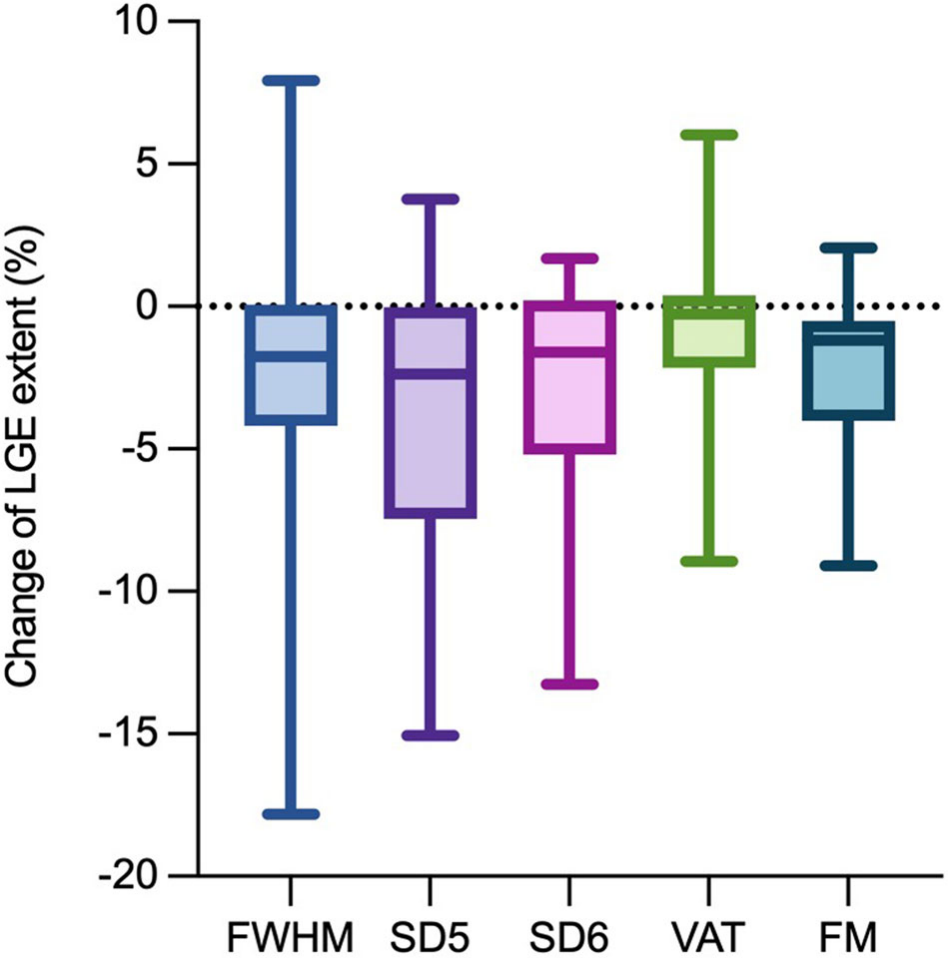
Absolute difference between LGE extent change among various quantitative techniques. Plots show LGE changes using different quantitative techniques, measuring LGE as a percentage (%) of left ventricular volume. Significant variability in LGE change, surpassing the ≥ 1% LV threshold, is evident. The largest difference is between SD5 and VAT techniques. Upper and lower lines represent max and min points, colored boxes enclose the first and third quartiles, with lines indicating medians

All patients exhibited nonischemic (subepicardial) LGE in at least one myocardial segment. The extent of LGE at baseline and follow-up, assessed with different techniques, is summarized in Fig. 3 and Table 3. Significant variation in LGE extent change was noted among quantitative techniques (*p* < 0.01), with SD5 showing the largest change (−2.4% [IQR: −7.2; −0.1%]) and VAT the smallest (−0.3% [IQR: −2.1; 0.3%]). Absolute change in LGE extent across techniques exceeded the predefined threshold of a significant change (≥ 1% of LV), leading to the disparate categorization of patients at follow-up, as demonstrated also in Fig. 4. Consequently, there was a significant disparity among LGE assessment techniques in categorizing patients with progressive, unchanged, or regressive LGE at follow-up. The highest proportion of patients with LGE change was identified with SD5 (60%), while the lowest was with VAT (34%), as summarized in Table 3.

**Table 3:** LGE assessment comparing baseline to the 3-month follow-up CMR

*n* = 47	Baseline LGE amount	Follow-up LGE amount	LGE extent change	Patients with LGE progression	Patients with LGE regression
**Quantitative**					
FWHM, median [IQR] or *n* (%)	3.7 [1.6; 6.5]	1.8 [0.7; 2.5]	−1.8 [−3.8; 0]	3 (6)	26 (55)
SD5, median [IQR] or n (%)	4.0 [1.8; 8.8]	2.1 [0.9; 3.8]	−2.4 [−7.2; −0.1]	7 (15)	28 (60)
SD6, median [IQR] or *n* (%)	2.7 [0.8; 6.8]	1.0 [0.4; 2.2]	−1.6 [−5.2; 0]	4 (9)	27 (57)
VAT, median [IQR] or *n* (%)	2.0 [0.7; 4.6]	1.5 [0.7; 2.4]	−0.3 [−2.1; 0.3]	4 (9)	16 (34)
FM, median [IQR] or *n* (%)	3.0 [1.4; 6.2]	1.6 [0.3; 2.4]	−1.2 [−3.8; −0.5]	2 (4)	26 (55)
**Semiquantitative**					
VPS, median [IQR] or *n* (%)	2 [1; 4]	1 [1; 2]	−1 [−1; 0]	0 (0)	25 (53)
VTS, median [IQR] or *n* (%)	5 [2; 9]	2 [1; 4]	−2 [−4; −1]	0 (0)	36 (77)
VCS, median [IQR] or *n* (%)			−3 [−4; −2]	0 (0)	42 (89)

Data are presented as median and interquartile range [IQR] or count and percentage (%), as appropriate

Baseline and follow-up LGE are expressed as percentages relative to the LV myocardium volume for quantitative methods and as scores for semiquantitative techniques. LGE extent change, indicated by negative values for a decrease and positive for an increase, reflects the difference in LGE amount between follow-up and baseline CMR. Progression and regression indicate the number of patients with LGE progression or regression at follow-up compared to baseline. For quantitative methods, a change of ≥ 1% in LV volume was considered significant, while semiquantitative techniques deemed any change in the scoring category as significant

**Fig. 5:**
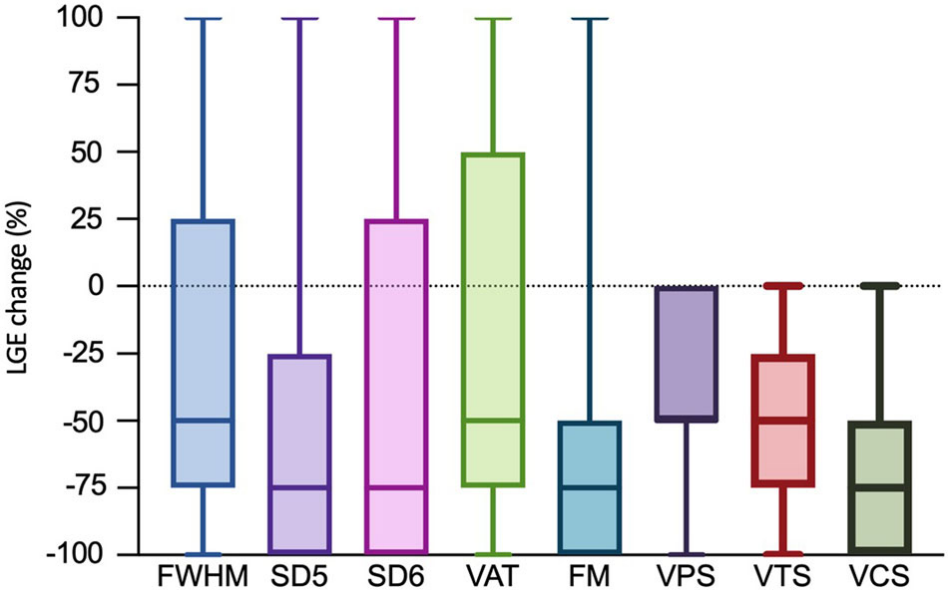
Relative changes in LGE extent during follow-up, illustrated for both quantitative and semiquantitative techniques in comparison to visual change score. Plots show LGE change from baseline to follow-up CMR, adjusted for visual change score. Data from quantitative and semiquantitative techniques was converted into VCS categories, as 1–25%, 26–50%, 51–75%, and 76–100% change. Positive values denote LGE progression, negative values indicate LGE regression. Upper and lower lines depict max and min points, while colored boxes indicate first and third quartiles, with lines showing medians

Semiquantitative grading showed LGE regression in 53% of the patients with VPS and 77% with VTS during follow-up. Visual assessment with VCS demonstrated higher sensitivity for detecting LGE extent change, with positive results in 89% of patients. Importantly, neither semiquantitative scoring nor visual assessment using VCS showed LGE progression between baseline and follow-up CMR. Figure 5 offers a comparative view of VCS with traditional assessment techniques regarding proportional LGE changes during follow-up.

### Repeatability of LGE assessment

Quantitative postprocessing methods showed excellent interobserver repeatability for LGE assessment at baseline and follow-up, with the highest ICC values observed for FHWM (up to 0.98 [IQR: 0.96; 0.99]), as summarized in Table 4. ICCs at follow-up CMR were slightly reduced for SD5 and SD6 (0.79 [IQR: 0.65; 0.87] and 0.78 [IQR: 0.64; 0.87], respectively), indicating potentially lower reliability for methods involving gray-scale thresholds in quantifying smaller amounts of LGE. The repeatability of assessing changes in LGE extent was excellent for most quantitative techniques, with FWHM having the highest ICC (0.97 [IQR: 0.94; 0.98]), and good for SD5 (0.89 [IQR: 0.81; 0.93]).

**Table 4:** Intraclass correlation coefficients among three observers for LGE assessment using different techniques

*n* = 47	Baseline	Follow-up	LGE change
**ICC quantitative**			
FWHM, median [IQR]	0.98 [0.96; 0.99]	0.98 [0.97; 0.98]	0.97 [0.94; 0.98]
SD5, median [IQR]	0.94 [0.90; 0.96]	0.79 [0.65; 0.87]	0.89 [0.81; 0.93]
SD6, median [IQR]	0.93 [0.89; 0.96]	0.78 [0.64; 0.87]	0.91 [0.86; 0.95]
VAT, median[IQR]	0.94 [0.89; 0.96]	0.95 [0.91; 0.97]	0.92 [0.87; 0.95]
FM, median [IQR]	0.95 [0.91; 0.97]	0.91 [0.86; 0.95]	0.92 [0.87; 0.95]
**ICC semiquantitative**			
VPS, median [IQR]	0.80 [0.59; 0.89]	0.66 [0.39; 0.81]	0.81 [0.63; 0.90]
VTS, median [IQR]	0.77 [0.52; 0.89]	0.89 [0.56; 0.90]	0.71 [0.53; 0.83]
CS, median [IQR]			0.93 [0.89; 0.96]

Data presented as median and interquartile range [IQR]. LGE change represent the difference between baseline and follow-up CMR

Interobserver repeatability of semiquantitative scoring was good for VPS at baseline (0.80 [IQR: 0.59; 0.89]), VTS at baseline (0.77 [IQR: 0.52; 0.89]) and follow-up (0.89 [IQR: 0.56; 0.90]), but moderate for VPS at follow-up (0.66 [IQR: 0.39; 0.81]). VPS change showed good interobserver repeatability (0.81 [IQR: 0.63; 0.90]), which was moderate for VTS change (0.71 [IQR: 0.53; 0.83]).

**Fig. 6:**
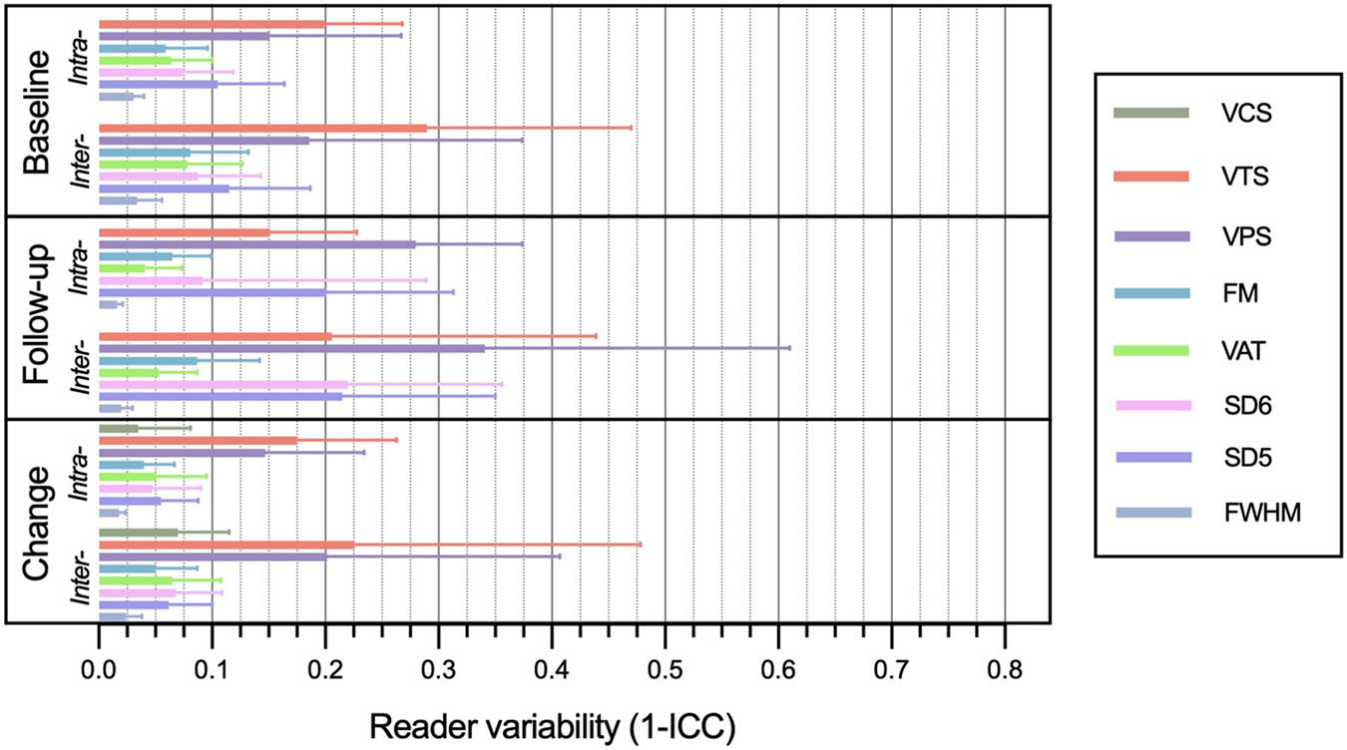
Repeatability of LGE assessment techniques. Intra- and interobserver repeatability of LGE assessment methods at baseline, follow-up, and for LGE extent changes presented as median and IQR for intraclass correlation coefficients (1-ICC)

Notably, VCS demonstrated excellent interobserver repeatability for assessing changes between baseline and follow-up (0.93 [IQR: 0.89; 0.96]). Inter- and intraobserver repeatability of various assessment techniques are summarized in Fig. 6.

### Association between different LGE assessment techniques

We found a positive correlation between visual scoring using VPS and VTS and all quantitative techniques, with a very strong association between FM and VTS (ρ = 0.73, *p* < 0.01), a moderate interrelationship between FWHM and VPS (ρ = 0.31, *p* = 0.03), and strong agreement between all other methods, as summarized in Table 5. Furthermore, VPS and VTS showed a strong association with VCS (ρ = 0.68 and ρ = 0.60, respectively, *p* < 0.01 both). We also observed a moderate positive association of VCS with VAT and FM (ρ = 0.35, *p* = 0.02 and ρ = 0.38, *p* = 0.01, respectively), but not with other quantitative techniques.

**Table 5:** The relationship among LGE assessment techniques in tracking changes during myocarditis follow-up

	VPS			VTS			VCS		
	ρ		*p* value	ρ		*p* value	ρ		*p* value
**FWHM**	0.31	0.03		0.40	0.01		0.26	0.08	
**SD5**	0.44	< 0.01		0.62	< 0.01		0.25	0.07	
**SD6**	0.45	< 0.01		0.60	< 0.01		0.27	0.07	
**VAT**	0.45	< 0.01		0.56	< 0.01		0.35	0.02	
**FM**	0.47	< 0.01		0.73	< 0.01		0.38	0.01	
**VPS**	–	–		–	–		0.68	< 0.01	
**VTS**	–	–		–	–		0.60	< 0.01	

The table outlines correlations between various LGE assessment techniques measuring changes in LGE extent from baseline to follow-up CMR. Correlation coefficients (ρ) and *p* values are presented, with significance set at *p* < 0.05

## Discussion

We demonstrated excellent repeatability with commonly used postprocessing software for quantitative tracking of LGE in acute myocarditis, with the least observer dependence observed with the FWHM technique. Semiquantitative scoring using VPS and VTS emerged as a reliable alternative for assessing LGE changes in myocarditis, showing a strong association with most quantitative methods, albeit with slightly lower overall repeatability compared to quantitative approaches. Our simplified method for a direct comparison of LGE throughout the entire LV myocardium, referred to as the visual change score, proved highly capable of detecting LGE alterations over time with excellent repeatability. Importantly, the categorization of patients’ LGE status at follow-up CMR (progression/regression/no change) showed substantial variation across LGE assessment techniques.

A recent meta-analysis of 11 studies revealed that extensive LGE at the index CMR doubles the risk of future cardiovascular events following acute myocarditis. However, comparing LGE burden across studies is deemed challenging due to varied definitions, such as LGE affecting more than 2 myocardial segments, 10% of the LV mass, or 17 grams, depending on the assessment technique [18]. This highlights the need for comprehensive studies. Gräni et al conducted an extensive study on 670 patients, demonstrating that the FWHM technique had the highest repeatability for baseline LGE assessment in myocarditis [13], consistent with our findings. Almost all quantitative techniques in our study showed excellent repeatability, with SD5 and SD6 remaining in the good range for the follow-up. Given the LGE decrease at the follow-up CMR in most patients in our cohort, it can be speculated that these threshold-based techniques may exhibit decreased interobserver reliability with smaller amounts of LGE. CMR is also increasingly used for ongoing disease monitoring. Prior longitudinal studies revealed that LGE decreases in up to a quarter of patients six months after the acute phase of myocarditis, indicating that LGE does not necessarily imply definite fibrosis [19]. In a recent study by Aquaro et al involving 187 patients with repeated CMR after acute myocarditis over a median clinical follow-up of seven years, an increase in LGE extent at the 6-month follow-up was associated with future cardiovascular events (HR 2.60, 95% CI 1.05–6.90). Importantly, even the persistence of LGE on follow-up CMR was an independent risk factor when concomitant resolution of myocardial edema was observed (HR 4.50, 95% CI 1.30–14.50) [6]. This underscores the importance of precise evaluation of LGE changes over time in myocarditis patients. However, quantitative analysis is often time-consuming, requiring segmentation of complete LV endo- and epicardial myocardium contours. Additionally, the use of various postprocessing methods at different time points during imaging follow-up may introduce errors, raising questions about the interchangeability of these techniques for longitudinal studies. Our findings highlight excellent reliability in assessing LGE changes in myocarditis when using the same quantification technique for the follow-up, with the FWHM showing the highest repeatability. Notably, the absolute difference in LGE change among various postprocessing techniques in our study exceeded the predefined threshold of ≥ 1% of the LV mass [6], indicating their lack of interchangeability for follow-up studies. Consequently, the proportion of patients classified with LGE progression, regression, or no change varied markedly depending on the postprocessing technique used, suggesting that the prognostic information derived from quantitative LGE analysis may strongly depend on the chosen technique. Semiquantitative LGE scoring with VPS and VTS is a reliable alternative to quantitative techniques, with slightly lower repeatability in our study. Nevertheless, there is a positive correlation between the performance of VPS and VTS and most quantitative methods in tracking LGE changes during follow-up. We also tested a simplified visual assessment method, referred as ‘visual change score’, for monitoring global LGE changes in the entire LV myocardium during follow-up. VCS was highly reproducible in our study for the direct comparison of LGE between baseline and follow-up CMR. Moreover, it exhibited a strong correlation with VPS and VTS, and a moderate correlation with FM LGE quantification and semiautomated LGE assessment using the VAT technique. Simplified visual analysis has been supported by evidence in cardiac imaging [20–22]. Even prior large-scale trials, such as the CE-MARC trial, have employed a visual approach to estimate wall motion abnormalities, ischemia testing, coronary stenosis, and infarct scar size [23]. This simplified, less time-consuming method may be particularly valuable in small centers where sophisticated and often more expensive postprocessing techniques are not available. However, the prognostic potential of VCS remains unknown, and further studies with clinical follow-up and repeated CMR are necessary, especially since we couldn’t establish a correlation of VCS with FHWM, SD5, and SD6 techniques, which have been the preferred techniques in prior prognostic studies using CMR in myocarditis follow-up [6, 10].

Our study has limitations. While our study is limited by a smaller sample size and a comparatively young median patient age, the use of CMR in diagnosing acute myocarditis, coupled with stringent exclusion criteria, has allowed for a more accurately defined cohort of acute myocarditis cases. This approach offers a clearer distinction than larger studies that depend only on clinical criteria [24]. We used a previously established 1% threshold for change in LGE volume [6], which may yield lower specificity in larger LGE volumes. An increased threshold for LGE extent change would also probably smoothen the difference between the quantification methods in regards of categorizing patients with changing or stable LGE. This was not further tested in our study, and the sensitivity and specificity of this threshold for true biological changes remain underexplored. Variations in hardware and software could still influence technique performance. Our sample size did not allow for comparisons between 1.5 T and 3 T scanners or various LGE sequences, but our previous research suggested similar diagnostic quality for 2D and 3D LGE techniques [25]. The lack of clinical follow-up limits our ability to determine the prognostic value of certain techniques, including VCS, which was not the primary focus of this study. Larger studies with clinical follow-up are needed to address these limitations.

In conclusion, our study highlights non-interchangeability among methodologies for tracking myocarditis fibrotic changes. Among common postprocessing techniques, FWHM shows the highest degree of repeatability of LGE assessment during follow-up in this patient population. Visual scoring with VPS and VTS is a reliable alternative, though with slightly lower repeatability. We introduced a simplified visual change score for direct LGE change comparison, revealing strong correlations with semiquantitative techniques. This approach is valuable for smaller centers with limited resources. Additional studies with clinical follow-up are necessary to determine its prognostic potential.

## Abbreviations

CMR: Cardiovascular magnetic resonance
FHWM: Full width at half maximum
FM: Full manual
ICC: Intraclass correlation
IQR: Interquartile range
LGE: Late gadolinium enhancement
LV: Left ventricle
SD5: Gray-scale thresholds set at 5 standard deviations above remote myocardium
SD6: Gray-scale thresholds set at 6 standard deviations above remote myocardium
T: Tesla
VAT: Visual assessment with threshold
VCS: Visual change score
VPS: Visual presence score
VTS: Visual transmurality score
